# Dysbiotic microbiota contributes to the extent of acute myocardial infarction in rats

**DOI:** 10.1038/s41598-022-20826-z

**Published:** 2022-10-03

**Authors:** Marc-André Gagné, Claude Barbeau, Geneviève Frégeau, Kim Gilbert, Olivier Mathieu, Jérémie Auger, Thomas A. Tompkins, Emmanuel Charbonney, Roger Godbout, Guy Rousseau

**Affiliations:** 1 Research Center, CIUSSS du Nord-de-l’Île-de-Montréal, Hôpital Sacré-Cœur, Montreal, Canada; 2 Lallemand Health Solutions, Montréal, Canada; 3 Centre de recherche du Centre Hospitalier de l’Université de Montréal (CHUM), Montreal, Canada; 4 Department of Psychiatry, Université de Montréal, Montreal, Canada; 5 Department of Pharmacology and Physiology, Université de Montréal, Montreal, Canada; 6 Centre de biomédecine, Hôpital du Sacré-Cœur de Montréal, CIUSSS du nord de l’île de Montréal, 5400 Gouin Boulevard Ouest, Montreal, QC H4J 1C5 Canada

**Keywords:** Cardiovascular biology, Myocardial infarction, Biochemistry, Physiology, Cardiology, Medical research

## Abstract

Increasing evidence suggests that the intestinal microbiota composition could play a role in specific pathologies such as hypertension, obesity and diabetes. This study aims to demonstrate that the intestinal microbiota modulated by a diet creating dysbiosis increased the size of the myocardial infarction and that probiotics could attenuate this effect. To do this, microbiota transplants from rats fed a dysbiotic or non-dysbiotic diet in the presence or absence of probiotics were performed for 10 days on rats whose microbiota had been previously suppressed by antibiotic therapy. Then, the anterior coronary artery of the transplanted rats was occluded for 30 min. Infarct size was measured after 24 h of reperfusion, while signaling pathways were evaluated after 15 min of reperfusion. Intestinal resistance, plasma concentration of LPS (lipopolysaccharides), activation of NF-κB and Akt and composition of the microbiota were also measured. Our results demonstrate a larger infarct size in animals transplanted with the dysbiotic microbiota without probiotics compared to the other groups, including those that received the dysbiotic microbiota with probiotics. This increase in infarct size correlates with a higher firmicutes/bacteroidetes ratio, NF-kB phosphorylation and plasma LPS concentration, and a decrease in intestinal barrier resistance and Akt. These results indicate that dysbiotic microbiota promotes an increase in infarct size, an effect that probiotics can attenuate.

## Introduction

Over the past decades, studies have shown a role for the intestinal microbiota in several physiological functions^1^. In addition to participating in the catabolism of certain substances, experimental results indicate that the microbiota may also contribute to the integrity of the intestinal barrier, the control of the immunological response, the prevention of the spread of pathogenic microorganisms, and the absorption and metabolism of certain nutrients. An imbalance in the composition of the microbiota can have significant consequences on the development or amplification of pathologies such as cardiovascular disease.

We have known for several decades that our diet can have an important effect on the development of cardiovascular diseases by increasing, among other things, triglycerides and cholesterol. However, despite this obvious link between diet and cardiovascular disease, the intestinal microbiota has been a neglected player. The intestinal microbiota can not only be altered by diet, but it may also contribute to cardiovascular disease by increasing the patient’s inflammatory state.

Diets may induce an “imbalance” in the gut microbial community, leading to dysbiosis. Dysbiosis has frequently been reported in patients with cardiovascular risks^2^. Certain types of high carbohydrate diets may increase Bifidobacteria^3^ while high-fat diets are associated with reduced Bacteroidetes, increased Firmicutes and increased Gram^−^/Gram^+^ ratio, leading to dysbiosis and ultimately to inflammation^4,5^.

Among other things, variation in the composition of the microbiota could alter the integrity of the intestinal barrier^6^. The layer of epithelial cells that constitutes the intestinal barrier makes it possible to form, with the layer of mucus and other types of cells, a physicochemical barrier that prevents the translocation of intestinal contents into the bloodstream. The presence of dysbiosis can induce the remodeling of this barrier, allowing the transfer of intestinal contents to the bloodstream, causing an increase in inflammation.

There is evidence, although limited, that the microbiota can influence the size of myocardial infarction. For example, drinking liquids containing the probiotic *Lactobacillus plantarum* 299v has been shown to reduce myocardial infarct size in rats^7^. Another study by Lam et al*.* has demonstrated an association between certain metabolites in the gut microbiota and the severity of myocardial infarction^8^. We have also reported that the integrity of the intestinal wall was altered after myocardial infarction and that this alteration was reduced in the presence of probiotics, which suggests a link between heart damage and the gut microbiota^9^. These results indicate that the probiotics may have beneficial effects on the integrity of the intestinal barrier in presence of dysbiosis.

Because inflammation influences the development of cardiovascular diseases, and because dysbiosis can contribute to inflammation, we hypothesised that microbiota from a dysbiotic (D) diet might increase infarct size compared to a non-dysbiotic (ND) diet. This effect could be attenuated in the presence of probiotics, in an attempt to restore balance.

To determine the effect of microbiota on myocardial infarct size, we proceeded to transplant microbiota isolated from D or ND donor rats (donor) ± probiotics in recipient rats, treated previously with antibiotics to remove commensal microbiota.

## Materials and methods

### Ethics statement

The experiments complied with the animal care guidelines published by the Canadian Council on Animal Care. The procedures performed (Grou57) were approved by the local Animal Care Committee of the Hôpital du Sacré-Coeur de Montréal. Our study experiments were performed in accordance with ARRIVE guidelines (Animal Research: Reporting of In Vivo Experiments)^10^.

### Experimental design

One hundred twelve adult Sprague–Dawley albino male rats (approximately 300 g) (Charles River, Saint-Constant, QC, Canada) were housed in individual cages at 22 °C with a relative humidity of 40%–50% and subjected to a 12 h light/dark cycle, starting at 8:00 a.m. The rats had three days of acclimatisation to their environment with a standard diet (Charles River). The rats were then randomly separated into two groups: 36 donor rats and 76 recipient rats.

The donor group was divided into 4 subgroups, and for a minimum of 21 days before collecting their faeces for faecal transplants and throughout the rest of the protocol, they were fed with a ND or D diet (Table 1) combined or not with probiotics dissolved in water (*Bifidobacterium longum R0175* and *Lactobacillus helveticus R0052*).

**Table 1:** Composition of the diets used in the study.

	Non-dysbiotic diet	Dysbiotic diet
**% diet weight**		
Protein	22.0	22.0
Carbohydrate	42.9	42.9
Fat	20.3	20.3
**Lipids (g/kg)**		
Saturated	78.1	71.3
Monounsaturated	33.7	39.7
Polyunsatured	83.2	88.2

In parallel, the recipient group was fed ad libitum with a standard diet (Charles River) throughout the study. The recipients received a mixture of antibiotics in their drinking water for 15 days to suppress their gut microbiota.

A transplant of the intestinal microbiota from the donor group to the recipient group was performed 3 days after the end of the antibiotic treatment, and this was done for 10 consecutive days. The recipient group was then divided into subgroups corresponding to the subgroups of the donor group: one group received the microbiota from a ND diet with vehicle; a second group received the microbiota from a ND diet with probiotics; a third group received the microbiota from a D diet with vehicle; and fourth group received the microbiota from a D diet with probiotics. After 10 days of transplantation, the recipient rats underwent cardiac surgery where the left anterior descending artery was occluded for 30 min. Some rats were sacrificed after 15 min of reperfusion to allow evaluation of signaling pathways. The remaining rats were sacrificed after 24 h of reperfusion and were used to evaluate myocardial infarct size, intestinal permeability, as well as plasma concentration of LPS.

### Diets

Animals from both donor groups received a specific diet rich in lipids (22% protein, 42.9% carbohydrates, 20.3% fat) from Envigo Teklab (Madison, WI, USA). The detail of the composition of the diets enriched in fat is presented in Table 1. All the recipient rats received a standard diet (Charles River).

### Probiotics

The blend of *Lactobacillus. helveticus* R0052 and *Bifidobacterium longum* R0175 was administered by suspending the freeze-dried culture or the vehicle only (maltodextrin) in 200 mL of tap water. Each rat in the probiotics group received a daily dose of 10^9^ colony-forming units. The drinking solution was freshly prepared each day for the duration of the experiments. Water intake was monitored throughout the investigation to ensure that a sufficient amount of bacteria was administered.

### Antibiotic therapy

Before microbiota transplantation, recipient rats received a mixture of antibiotics in their drinking water for 10 days (ampicillin 1.25 g/L, Aurobindo; imipenem/cilastatin 350 mg/L, Sandoz; vancomycin 600 mg/mL, Sandoz; metronidazole 60 mg/L, Hospira; ciprofloxacin 40 mg/L, Sandoz) in order to suppress their intestinal microbiota as described in Kelly et al.^11^ Antibiotics in powder form (ampicillin, imipenem, and vancomycin) were dissolved in 5% dextrose beforehand. Water intake was monitored daily to ensure that a sufficient amount was consumed.

### Preparation of the gut microbiota

The gut microbiota solutions were prepared according to an established technique^12^ and recommended by a European consensus^13^. Fresh faeces from the same animals (6 rats/diet) were pooled, homogenised in 10 ml of sterile phosphate-buffered saline, and centrifuged for 10 min at 4 °C. The supernatants’ optical density values were calculated and diluted to obtain 1 × 10^10^ bacteria/500 µl. This preparation was freshly prepared, added daily to gelatin (10 ml), and given to recipients.

### Surgical procedures

Anesthesia was induced by ketamine xylazine (80 mg/kg and 10 mg/kg i.p., respectively) and maintained with isoflurane (1%). The animals were intubated and placed on a respirator. The left thoracotomy was undertaken, and the left anterior descending coronary artery was occluded with a 4.0 silk suture and plastic snare. Ischemia was assessed with ST-segment alterations and ventricular subepicardial cyanosis. The suture was removed after 30 min of ischemia for reperfusion. The thorax was closed, and the rats were injected with analgesics (0.05 mg/kg of butorphanol sc)^14^. After 15 min or 24 h of reperfusion, decapitation was used for sacrifice to prevent biochemical pathway modifications.

### Infarct size measurement

The hearts were removed immediately after euthanasia and placed in dishes kept on crushed ice. They were washed with saline by retrograde perfusion via the aorta. The left anterior descending coronary artery was occluded at the same site as for MI induction (see above) to map the area at risk (AR) by Evans blue infusion (0.5%). The hearts were frozen (− 80° C for 5 min), sliced into 4 transverse 2 mm sections and placed in 2,3,5-triphenyltetrazolium chloride solution (1%, pH 7.4) at 37° C for 10 min to better distinguish the area of necrosis (I) from the AR. The different regions were carefully drawn on a glass plate, photocopied, and cut. Thereafter, the complete infarct region, AR and left ventricle (LV), were weighed separately to express MI as percentages of necrosis (I) of the AR (I/AR × 100), and AR as percentages of the LV area (AR/LV × 100).

### Biochemical analysis

#### Plasma LPS concentration

Plasma samples were prepared in MAPK buffer (20% Mix 5X pH 7.5 (50 mM Tris–HCl, 20 mM β-glycerophosphate, 20 mM sodium fluoride, 5 mM EDTA, 10 mM EGTA, 1 mM Na_3_VO_4_), 1.6 mM benzamidine, 2.5 M PMSF, 1 M leupeptin, 5 M DTT, 1 M microcystin, 100 M Triton X-100), and a final concentration of 200 μg of protein was added in an ELISA plate. The measurements were taken in accordance with the instructions provided by the supplier (Rat LPS ELISA kit, MyBioSources [cat: MBS268498]).

### Ex vivo intestinal resistance

Small intestine resistance was measured with a Ussing Chamber^15^. Using a 5 cm segment of jejunum, the mucosa was bluntly stripped from the seromuscular layer and a 1 cm^2^ of mucosa was placed in the cassette. Intestinal transepithelial resistance was measured continuously during 90 min. Chambers were continuously oxygenated to prevent cell death. Results at 90 min were reported for comparisons.

### Western blotting: Akt, NF-kB

Western blot analysis was performed as described previously^16^. Briefly, tissues were homogenised by sonication in lysis buffer (1% Triton X-100, 0.32 mol/L sucrose, 10 mmol/L Tris (pH 8.0), 5 mmol/L EDTA, 2 mmol/L DTT, 1 mmol/L PMSF, 10 mg/mL leupeptin, 10 mg/mL Pepstatin A, 10 mg/mL aprotinin). The tissue homogenates were incubated for 30 min at 4 °C and centrifuged at 10 000* g* for 15 min. Protein concentrations of the supernatant were quantified by the Lowry method. Aliquots of 100 µg protein were loaded in polyacrylamide gels (10%–15%) and migrated at 150 V for 75 min in a mini-gel apparatus (BioRad Laboratories, Hercules, CA, USA). Proteins were transferred to nitrocellulose membranes with a Trans-Blot semi-dry transfer cell (BioRad Laboratories). Using SNAP i.d. 2.0 system (Millipore, Etobicoke, ON, Canada), non-specific sites were blocked for 20 min incubation in Odyssey blocking buffer (Li-CoR, Lincoln, NE, USA) (1:1 with PBS). After PBS washing, membranes were incubated 10 min with primary antibody 1:1000 phospho-Akt (S473) (Rabbit Ab Cell signalling, Whitby, ON, Canada), total Akt (Rabbit Ab Cell signalling), NF-κB (Rabbit Ab Cell signalling) and phospho NF-kappaB p65 (Rabbit Ab Cell signalling). After washing, the membranes were incubated 10 min with secondary antibody 1: 15 000 (anti-rabbit IRDye 800CW Li-Cor). After washing, membranes were scanned with Odyssey LI-COR Clx, and band intensities were analysed with Image Studio. The same membranes were placed in stripping buffer (0.1 mol/l glycine, 1% SDS, pH 2.0, 1 h at room temperature) and reused with the same technique for ratio determination phosphoAkt/total Akt, and phosphoNF-κB/total NF-κB.

### Microbiota composition

Stools from recipient rats were collected at the moment of the sacrifice and stored at − 80 °C. Total DNA was extracted from stool samples using the QIAmp Fast DNA Stool miniKit from Qiagen Sciences (Germantown, MD, USA). The gut microbiota composition was determined using 16S amplicon sequencing following Illumina 16S Metagenomic Sequencing Library Preparation. Briefly, template gDNA was amplified with 16S universal primers targeting the V3–V4 regions (forward: 5′-CCTACGGGNGGCWGCAG-3′; reverse 5′-GACTACHVGGGTATCTAATCC-3′). Following the described 25 cycles of amplification, amplicons were visualised on a 2% agarose 96-wells precast gel (Invitrogen, G72080). The PCR products were purified with Agencourt AMPure beads (Beckman Coulter, A63881). Five µL of the amplicon PCR reaction was used as template for 8 cycles Index PCR using Illumina barcode indexes Nextera XT V2 primer set (Illumina, FC-131-2001). Another round of Ampure Beads purification was done following Index PCR. The purified PCR reactions were quantified with Quant-iT PicoGreen dsDNA assay (Life Technologies, P7589) on a fluorescent plate reader. A 200 ng PCR reaction quantity was taken from each sample and pooled into a single tube that was quantified with QuBit Broad Range assay (ThermoScientific, Q32853) according to manufacturer’s instructions. The pool was diluted to 1 ng/µL and quality controlled by running a High Sensitivity D1000 TapeStation assay (Agilent, 5067-5584/5585). The library was denatured with 0.2 N NaOH and loaded at a 14 pM concentration. Sequencing was performed on a MiSeq platform using V3 chemistry kit with 2 × 250 bp reads.

### Statistical analysis

The results are expressed as mean ± SEM. The number of animals was calculated based on a range of effect size of 30% from previous studies, alpha error of 0.05, and power of 0.8. Statistical analysis was performed using a 2-way ANOVA with Diet and Probiotics as independent variables. When the interaction was significant, a decomposition was performed to detect differences in the simple effect. Simple linear regression analysis was performed between the ratio of Firmicutes/Bacteroidetes and infarct size, expressed as a percent of the area at risk, or LPS to assess potential correlations between those factors. Analysis was performed using SPSS v. 26, and p < 0.05 values were considered to be significant.

## Results

Hemodynamic data indicated that the heart rate was stable during the experiment for the different groups (Table 2). On the other side, the mean arterial pressure decreased significantly in the four groups during the experiment, as compared to before occlusion,. However, no significant difference was observed between groups throughout the experiment eliminating the cardiac work as a factor to explain the difference in other measurements.

**Table 2:** Hemodynamic data.

	Non-dysbiotic	Non-dysbiotic + probiotics	Dysbiotic	Dysbiotic + probiotics
**Before occlusion**				
HR	218.6 ± 6.8	232.3 ± 10.4	222.1 ± 10.4	225.1 ± 13.4
MAP	73.7 ± 6.2	74.3 ± 6.9	91.5 ± 12.6	81.8 ± 10.9
**20 min occlusion**				
HR	219.1 ± 10.7	221.6 ± 7.3	226.6 ± 6.4	219.9 ± 12.6
MAP	61.9 ± 7.4*	49.7 ± 1.8*	71.0 ± 7.1*	54.8 ± 6.4*
**Reperfusion**				
HR	230.4 ± 15.2	212.3 ± 6.9	220.4 ± 6.4	204.9 ± 7.0
MAP	63.0 ± 8.6*	50.8 ± 2.3*	55.2 ± 7.1*	55.8 ± 8.2*

*HR* heart rate (beats/min), *MAP* mean arterial pressure (mm Hg).

*p < 0.05 compared to before occlusion values.

### Infarct size

**Figure 1:**
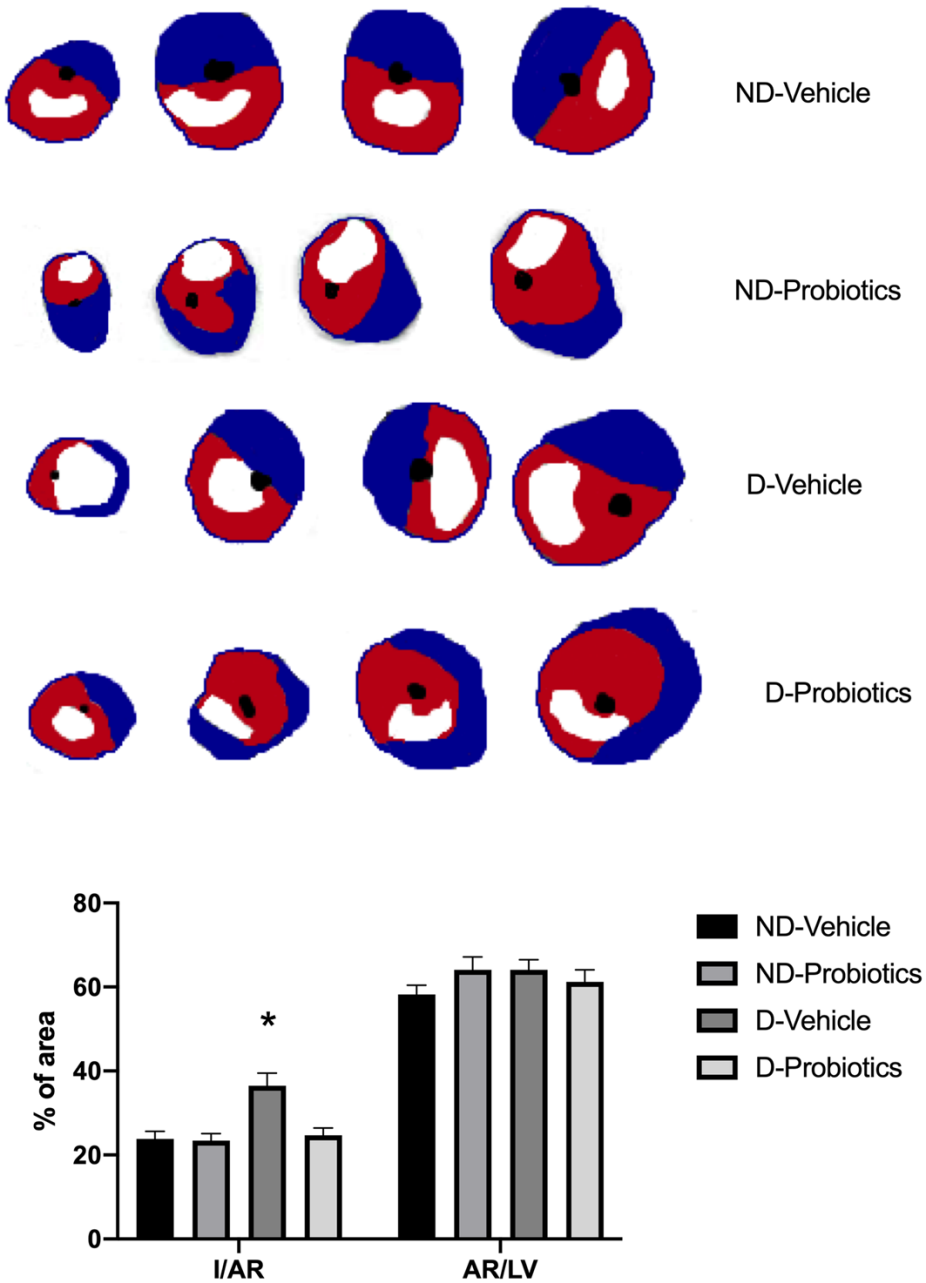
Upper panel: Representative images of myocardial infarction observed in the different groups (Blue: Evans Blue positive, normal region, White: TTC positive infarction, Red: TTC negative, Red and White represent AR). Lower panel: Infarct size (I) expressed as a percentage of the area at risk (AR), and AR as a percentage of the left ventricle (LV) after 24 h reperfusion. *p < 0.05 versus ND-vehicle and D-probiotics. *ND* none-dysbiotic, *D* dysbiotic.

Our results indicate that the dysbiosis-vehicle animals presented a 50% larger infarct size, expressed as a percent of the area at risk, as compared to the animal receiving the ND diet microbiota (p < 0.05). This increase in infarct size in the dysbiosis-vehicle group was prevented with probiotics indicating that microbiota participate to the progression of the infarct size in these conditions (Fig. 1).

Area at risk, expressed as percent of the left ventricle, are similar among groups (p > 0.05).

### Firmicutes/bacteroidetes ratio

**Figure 2:**
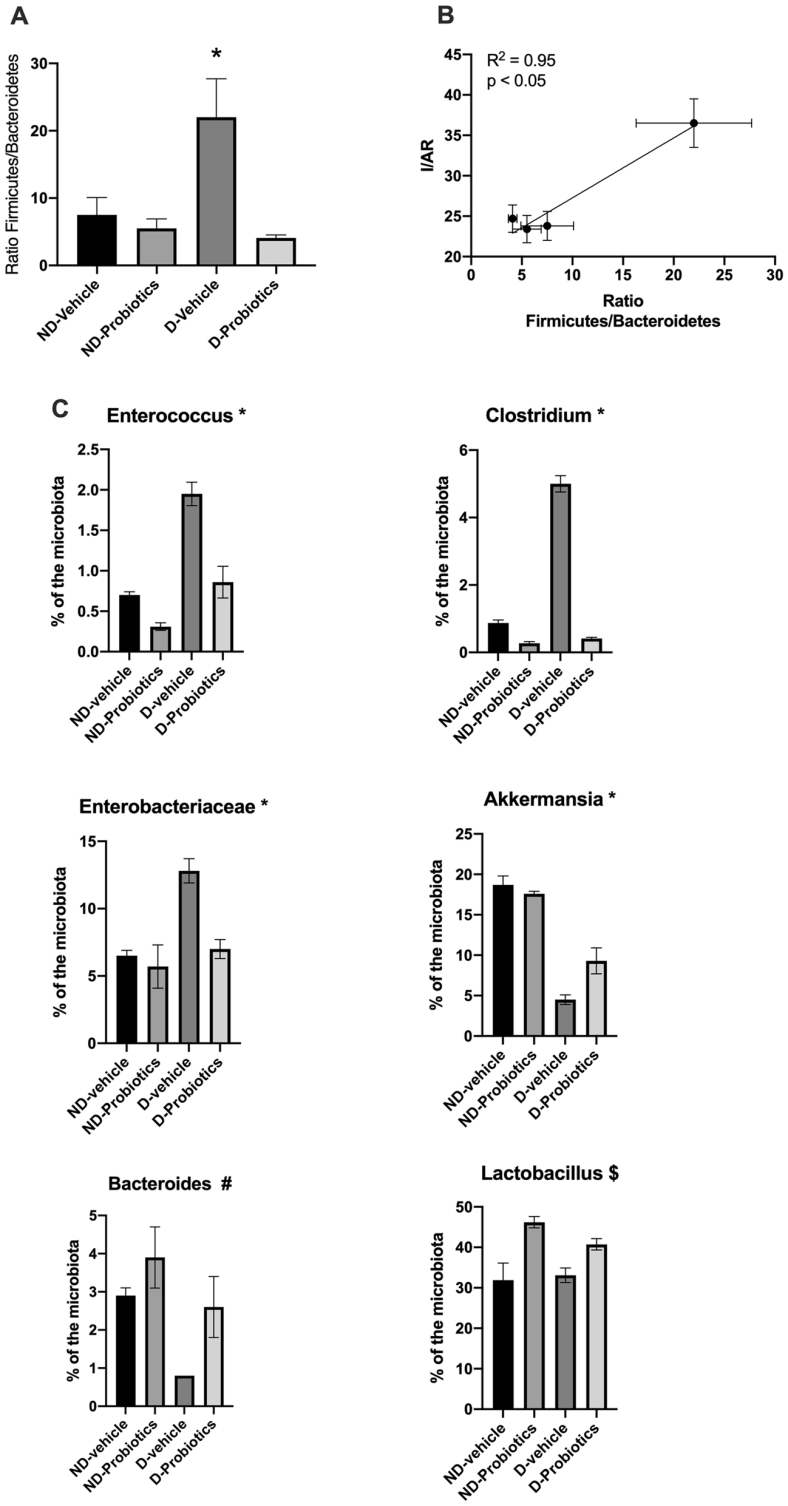
(**A**) Ratio of the Firmicutes/Bacteroidetes in the different recipient experimental groups *p < 0.05 versus ND-vehicle and D-probiotics. (**B**) Correlation between the ratio of Firmicutes/Bacteroidetes and infarct size expressed as a percent of the area at risk. To perform the regression, and since the measure were not performed in the same animals, we used the mean of the ratio and the infarct size for each group. (**C**) Relative abundance of different family/genus in the microbiota of the recipient animals at the time of sacrifice. *indicates a significant interaction in the analysis (p < 0.05), $ indicates a significant effect of the probiotics (p < 0.05) whereas # indicates a significant effect of the diet and probiotics (p < 0.05).

Firmicutes/Bacteroidetes ratio indicated a significant interaction between groups (F_(1,19)_ 6.36; p < 0.05). Simple effects of this interaction indicated that the ratio in the D-vehicle group was significantly higher compared to the other groups. Probiotics alone reduced the ratio (F_(1,19)_ 9.96; p < 0.05) as well as the ND diet compared to the D diet (F_(1,19)_ 4.36; p = 0.051 (Fig. 2A).

Interestingly, the relation between the infarct size and the Firmicutes/Bacteroidetes ratio indicated a strong relationship between both variables (r^2^ = 0.95, p < 0.05), giving another argument for a role of the microbiota on myocardial infarct size (Fig. 2B).

### Composition of the microbiota

Figure 2C shows the percent of some family/genus observed in the microbiota isolated from the recipient rats at the time of the sacrifice. In four of them (*Enteroccocus*, *Clostridium*, *Enterobacteriaceae,* and *Akkermensia*), we observed a significant interaction (p < 0.05) between diet and probiotic. The percent of *Enteroccocus*, *Clostridium,* and *Enterobacteriaceae* was significantly more important (p < 0.05) in the D-vehicle group as compared to the other groups, while *Akkermensia* was significantly less abundant (p < 0.05). The relative abundance of the bacteroides was higher in the ND diet (p < 0.05) as compared to the D diet and more important in presence of probiotics, independently of the diet (p < 0.05).

We also observed that the relative abundance of the *Lactobacillus* was increased in the probiotics groups as compared to the vehicle groups.

### LPS plasmatic concentrations and intestinal resistance

**Figure 3:**
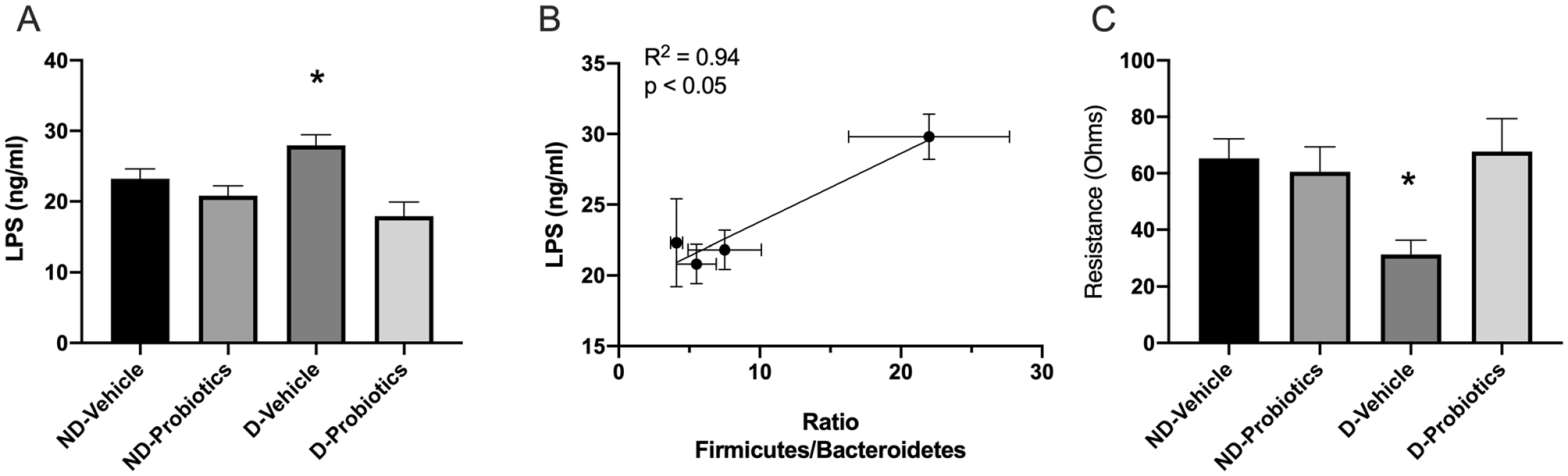
(**A**) Plasmatic concentrations of LPS in the different groups. *indicates a significant interaction between the main factors (Diet and Probiotics; p < 0.05). (**B**) Correlation between the ratio of Firmicutes/Bacteroidetes and the plasmatic concentrations of LPS (ng/ml). To perform the regression, and since the measure were not performed in the same animals, we used the mean of the ratio and the infarct size for each group. (**C**) Gut barrier resistance (ohms) measured in the different groups with the Ussing Chambers. *p < 0.05 versus ND-vehicle and D-probiotics. *ND* none-dysbiotic *D* Dysbiotic.

Because dysbiosis is associated with an increased in plasmatic concentration of LPS, we measured the concentrations of LPS in the different groups. The analysis indicated that the interaction between diet and probiotics was significant F_(1,27)_ = 4.87, p < 0.05. The simple effect indicated that the concentration observed in the D-vehicle was significantly higher as compared to the concentrations observed in the ND-vehicle or D-probiotics (p < 0.05). We also observed that probiotics lowered the plasmatic concentrations (F_(1,27)_ = 12.56, p < 0.05) (Fig. 3A). A correlation between the ratio of Firmicutes/Bacteroidetes found in the faeces and the plasmatic levels of LPS was found (Fig. 3B).

Since LPS can be translocated from the gut to the circulation, we suspected that the gut permeability could have been altered. Measuring the colon resistance, we observed a significant interaction between diet and probiotics (F_(1,25)_ = 5.22, p < 0.05). Simple effects analysis indicated that the resistance was lowered in the D-vehicle as compared to the other groups. Once again, the presence of probiotics in the D diet, results in a resistance that was similar to the one measured in the receiving ND diet, independently of the addition of probiotics (Fig. 3C).

### Akt and NF-κB

**Figure 4:**
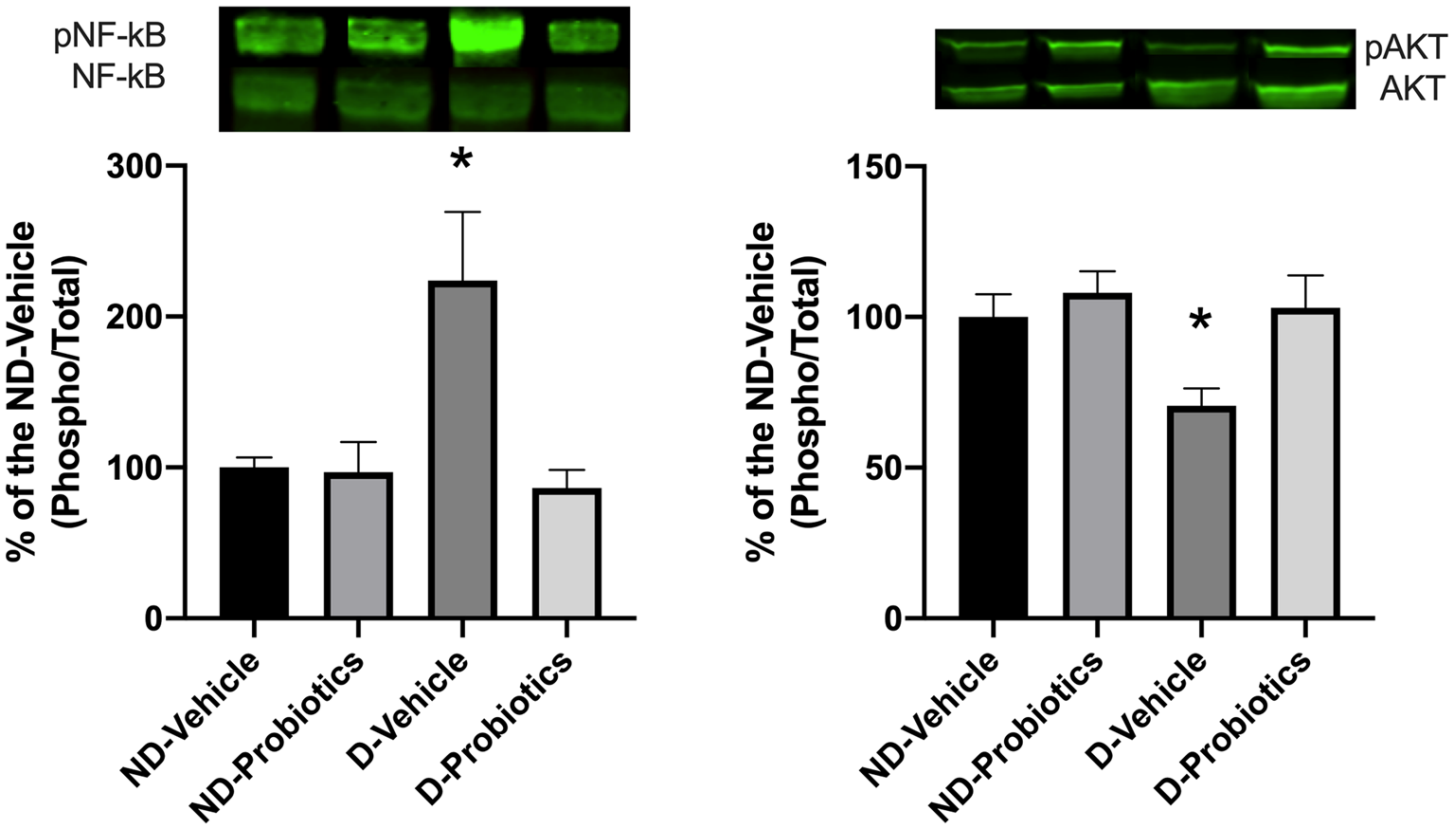
Left panel: p-NF-kB/NF-kB ratio right panel: p-Akt/Akt ratio. Results are expressed as a percentage of the ND-vehicle group in the myocardial ischemic region assessed by in vitro western blotting after 15 min of reperfusion. * indicates a significant interaction between the main factors (p < 0.05; Diet and Probiotics). Bands are cropped from original western blots presented in supplementary information file: Fig. 1A,B.

Two signaling pathways, Akt and NF-kB, known to influence the evolution of infarct size, were investigated to determine whether the modulation of the microbiota could alter their activation (Fig. 4). Significant interaction between diet and probiotics was observed (F_(1,20)_ = 4.88, p < 0.05) and simple effects indicate that the phosphorylation of NF-kB is higher in the D-vehicle group as compared to the other one (Fig. 4; Left).

No significant interaction was observed for Akt phosphorylation between the main factors (Diet and Probiotics), but we observed a higher level of phosphorylated Akt in the ND-diet group compared to the D-diet (F_(1,21)_ = 4.0.65, p < 0.05) whereas the presence of probiotics induced a higher level of phosphorylation of AKT compared to the vehicle (F_(1,21)_ = 6.42, p < 0.05; Fig. 4, Right).

## Discussion

The present study indicates that the microbiota isolated from rats on a dysbiotic diet has a deleterious effect on the ischemic myocardium resulting in an increase of infarct size as compared to a non-dysbiotic diet. The addition of probiotics to this dysbiotic diet altered the presence of the different bacteria family/genus, reversed the loss of the integrity of the intestinal barrier and the plasmatic concentrations of LPS, attenuated the activation of the inflammatory pathway (NF-kB), increased the activation of the enzyme involved in a salvaged pathway (Akt), and finally resulted in a reduction of myocardial infarction size.

High-fat or high-carbohydrate diets in humans induce dysbiosis characterised by a decrease in *Bacteroidetes* and an increase in *Firmicutes*^17^, which is also reported in rodents^4,5^. Our dysbiotic diet was elevated in fats and also resulted in an increase in the Firmicutes/Bacteroidetes ratio. It is interesting to note that both diets used in the present study were elevated in fats, suggesting that it was not only the content of fats that was important in inducing dysbiosis but also its composition. The major difference between the diets used was the omega-3/omega-6 fatty acids ratio, which was lower in the dysbiotic diet. However, since the quantities of the different lipids vary between diets, it would be important to determine the impact of the different lipids in the development of dysbiosis.

It was also reported that a high-fat diet results in an increase in the Gram^−^/Gram^+^ bacteria ratio^18^. The increase in Gram^−^ bacteria correlates with an increase in LPS, which promotes inflammation, as we also observed in the present study. We have also observed that the levels of different genera were increased in the dysbiotic diet without probiotics, such as *Clostridium, Enterococcus,* and *Eubacterium*, whereas *Akkermensia* was reduced. Some species of the first three families/genera are associated with an increase of inflammation although not in all of the species^19–21^, whereas some species associated with *Akkermensia* exhibit some anti-inflammatory properties^22,23^. However, we admit that our level of identification of the microbiotas was not sufficiently accurate to confirm this interesting hypothesis.

We also observed that the harmful effect of dysbiosis could be reversed by the addition of probiotics. The blend of probiotics that we used was previously shown to reduce apoptosis in the limbic system after myocardial infarction, while it was unable to reduce infarct size^24^. The rats used in this previous study were on a normal diet and without dysbiosis, as opposed to the present study^9,25^. Without dysbiosis, probiotics seem to have a minimal effect on the ischemic myocardium whereas, they attenuate the deleterious myocardial effect of dysbiosis in the presence of dysbiosis. Nevertheless, we must be aware that probiotics do not have the same effects, and it is important to target the mechanism we want to modify to choose the right probiotic or blend.

Although the design of the present study was not to determine the mechanism by which dysbiosis is deleterious for the ischemic myocardium, our convincing results of its impact might guide future research to identify the mechanisms involved.

Our findings point toward an increase in inflammation in the presence of dysbiosis, which can affect the size of the myocardial infarction. Many strategies have been elaborated to prevent or reduce inflammation during I/R, including the blockade of cytokines/chemokines, adhesion molecules, neutrophil depletion, and the administration of anti-inflammatory molecules^26–33^. Although not all of these have been successful, there are enough studies indicating that inflammation contributes negatively to myocardial infarction. Here we found that the activation of the myocardial NF-kB, a marker of inflammation, was greater in the dysbiotic group than in the other groups.

The inflammation observed in the dysbiotic group could be due to different factors, among which the measured elevation of plasmatic concentrations of LPS^34^. We have observed that the ratio of Firmicutes/Bacteroidetes in faeces correlated with the plasmatic concentrations of LPS. At this point, our results point toward a loss of the intestinal barrier integrity in the dysbiotic group, revealed by the reduced membrane resistance observed in the D-vehicle group. The increased permeability of the barrier could lead to bacterial product translocation and the initiation of an inflammatory response^35,36^, as pointed out by the increased circulatory LPS.

Regarding the protecting effect of probiotics on dysbiosis, some elements of the literature are worthwhile mentioning. For example, bacteria and their metabolites could also participate in the protective modulation of the infarct size by producing short chain fatty acids (SCFA), through their anti-inflammatory properties^37,38^. Non-digested polysaccharides are fermented by gut microbes, generating SCFAs, mostly acetate, propionate, and butyrate^39,40^, that could be beneficial for the myocardium, although this possibility is debated^41^. These metabolites present well-characterised anti-inflammatory properties and modulate cellular functions through G-protein coupled receptors (GPR41, GPR43, and GPR109A) or inhibiting histone deacetylases^42–45^.

Akt is among the different enzymes that are involved in the Reperfusion Injury Salvage Kinase (RISK) pathway, conferring cardioprotection and resulting in a reduction of infarct size when activated at the onset of reperfusion^46,47^. Lam et al. observed that the cardioprotection observed with vancomycin is lost in presence of the inhibition of Akt/PI3Kinase pathway and concluded that the gut microbiota metabolites are involved in the severity of the myocardial infarct size^8^ through the modulation of different signaling pathways.

Other mechanisms could also be involved. For example, it is suggested that the microbiota could contribute to cardiovascular disease through the production of phenylacetylglutamine (PAGIn)^48^. PAGln enhances platelet activation and thrombosis potential, leading to the development of myocardial infarction. However, MI was induced mechanically, and this aspect was not studied, but it remains that it could have contributed to our results.

The present study focused on the acute phase of myocardial infarction, but there are also recent reports on the effect of the microbiota on heart failure progression. This is an interesting avenue because heart failure develops a few weeks after the acute phase of myocardial infarction. Given the present results, one could hypothesize that a dysbiotic diet contributes to the development of heart failure.

Some limitations in the present study deserve to be mentioned. Since we observed different effects of the transplanted microbiota on myocardial infarct size, we now believe that the donor microbiota could have differed according to the diet used, but this measure was unfortunately not planned from the start. It has been reported that the effect of high fat diets on the Firmicutes/Bacteroidetes ratio is similar to what was found here in recipient rats^49,50^, suggesting that the diets may have indeed altered the composition of the donor’s microbiota. Also, the effect of the antibiotic treatment itself was not assessed. However, since all animals were treated the same across groups, we believe that the impact of the antibiotic treatment was similar. Therefore future studies will aim to characterize the donors’ microbiota, and the potential impact of the antibiotic treatment on recipient rats.

Overall the present study shows that a dysbiotic diet may contribute to the extent of myocardial infarction, for which the addition of probiotics could be protective. Dysbiotic microbiota could be harmful by affecting the integrity of the gut barrier promoting an inflammatory state, and precipitating the development of cardiovascular diseases and infarct size.

## Supplementary Information

Supplementary Figure 1.

## Data Availability

The datasets used and/or analyzed during the current study are available from the corresponding author upon a reasonable request as assessed by the Animal Research Ethics Committee of the corresponding author’s research center.
