# Supplementary material for: Dysbiotic microbiota contributes to the extent of acute myocardial infarction in rats
Source: Sci Rep. 2022 Oct 3;12:16517. doi: 10.1038/s41598-022-20826-z (PMC9530207; doi:10.1038/s41598-022-20826-z)
Supplement: Supplementary file 1 — Supplementary Figure 1. [file 41598_2022_20826_MOESM1_ESM.docx]

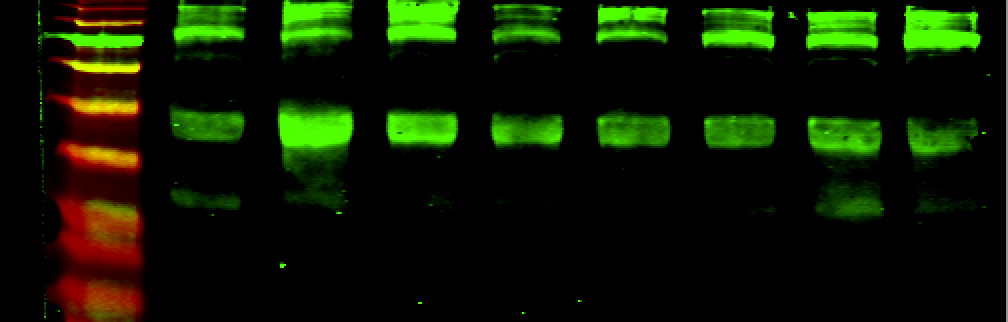

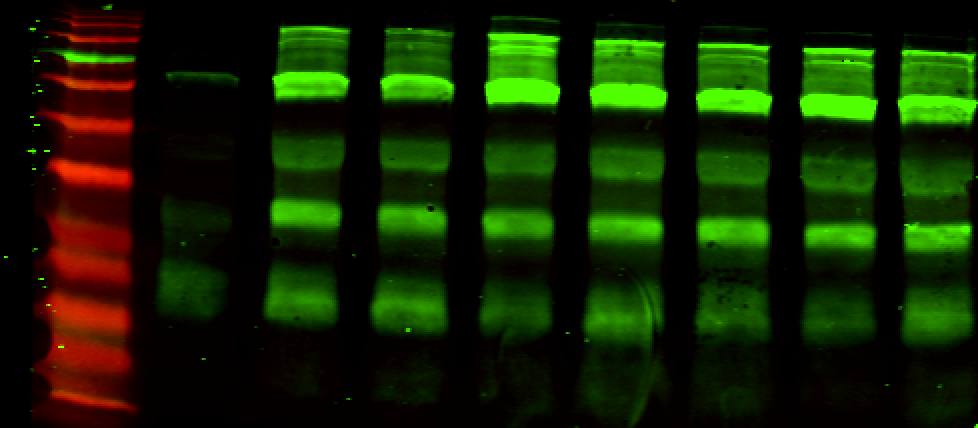


NF-κB

pNF-κB

MW ND-V D-V ND-P D-P ND-V D-P ND-P D-P

**Supplementary Figure 1A- Representative western blot of pNF-κB/NF-κB**

ND-V non-dysbiotic vehicle, ND-P non-dysbiotic probiotics

D-V dysbiotic vehicle; ND-P non-dysbiotic probiotics;

MW : Molecular weight


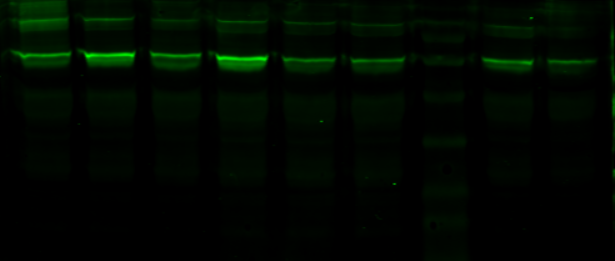

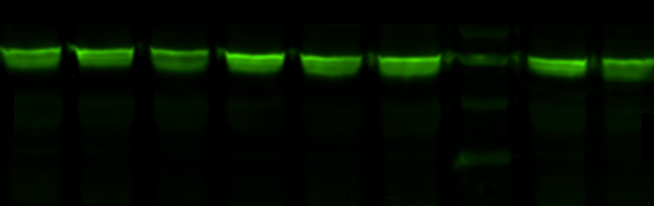


ND-V ND-P D-P D-P D-V D-V MW ND-V ND-P

pAKT

AKT

**Supplementary Figure 1B- Representative western blot of pAkt/Akt**

ND-V non-dysbiotic vehicle, ND-P non-dysbiotic probiotics

D-V dysbiotic vehicle; ND-P non-dysbiotic probiotics;

MW : Molecular weight
